# Old Age Psychiatry and Prison

**DOI:** 10.1192/j.eurpsy.2022.121

**Published:** 2022-09-01

**Authors:** V. Tort, J. Perez-Pazos, E. Perez-Sanchez, J.M. De Gomar-Malia, M. Peraire-Miralles

**Affiliations:** 1 Parc Sanitari Sant Joan de Deu, Forensic Psychiatry, Sant Esteve Sesrovires, Spain; 2 Hospital de Castelló, Forensic Psychiatry, Castello, Spain

**Keywords:** Prison, mental health, Old-age psychiatry, Inmates

## Abstract

Old age population is growing steadily during last decades (WHO 2017). Old population suffer from more morbidity including mental disorders (De Lorito,2018). This fact also applies for prison population (Yortons 2006) and in the lasts years has been an increasing interest for this topicThe aging of the world population is reflected in the penitentiary setting, with a progressive increase of elderly inmates. These prisoners present complex clinical processes with multiple comorbidities, requiring a specialized approach. In the 2020, the old age population in prison (considered over 60 years old) is around 5% in Catalonia (377 inmates,), same proportion (5%) in Spain.If we consider the cut-off point 50 y.o. (as other research) the proportion is 12% Our aims are to describe sociodemographical and clinical characteristics of old age inmates (over 50 y. o.) who required to be admitted to psychiatric unit in Catalan prisons between 2016 and 2020. The asample of this study will be around 150 inmates admitted in our unit. Elderly inmates present a high prevalence of substance abuse (especially alcohol), affective symptoms (depression) and cognitive deterioration. Likewise, is observed a significant presence of personality disorders, anxiety, post-traumatic disorders, psychotic disorders, and physical comorbidities. Prisoners over 50 have a different profile from the rest of the prison population. They suffer more physical and mental illnesses, so they require specific health and social approach. It would be advisable to adapt clinical care by optimizing resources, developing prison psychogeriatrics and establishing specific evaluation and treatment method

**Disclosure:**

No significant relationships.

